# Therapeutic implications of the tumor microenvironment in ovarian cancer patients receiving PD-1/PD-L1 therapy

**DOI:** 10.3389/fimmu.2022.1036298

**Published:** 2022-10-20

**Authors:** Yusha Wang, Lei Zhang, Yun Bai, Li Wang, Xuelei Ma

**Affiliations:** ^1^ Division of Biotherapy, Cancer Center, West China Hospital, Sichuan University, Chengdu, China; ^2^ Lung Cancer Center, West China Hospital, Sichuan University, Chengdu, China; ^3^ Department of Obstetrics and Gynecology, Chengdu First People’s Hospital and Chengdu Integrated Traditional Chinese Medicine (TCM) and Western Medicine Hospital, Chengdu, China; ^4^ West China School of Medicine, West China Hospital, Sichuan University, Chengdu, China

**Keywords:** tumor microenvironment, immunotherapy, immune cells, stromal cells, combination therapy

## Abstract

Epithelial ovarian cancer (EOC) ranks as the second most common cause of gynecologic cancer death. The conventional treatment for patients with EOC is postoperative therapy along with platinum chemotherapy. However, a more efficient treatment regimen is of great need for these patients diagnosed with advanced disease (FIGO stages III–IV), whose survival is approximately 29%. Immunotherapy seems to be an encouraging therapeutic strategy for EOC. Given the crucial role in the complicated interactions between tumor cells and other cells, the tumor microenvironment (TME) influences the response to immunotherapy. In this review, we discuss feasible strategies for EOC immunotherapy by exploiting the reciprocity of cancer cells and the constituents of the TME.

## 1 Introduction

Epithelial ovarian cancer (EOC) recognized by its high occurrence and poor prognosis (1), ranks as the second most common cause of gynecologic cancer death (2). The conventional treatment for EOC patients is postoperative therapy along with platinum chemotherapy (3). However, survival is dismal since over two-thirds of patients are diagnosed with advanced disease (FIGO stages III–IV) (4), and the survival rate for advanced stages is about 29% (5). Thus, a more effective treatment is of great need for these patients. Currently, immunotherapy is an encouraging treatment for various cancers (6). Immunotherapy agents are used to activate effector and cytotoxic T cells that respond to cancer cells through natural mechanisms, many of which are suppressed during cancer progression (7). Poor response to immunotherapy in ovarian tumors was associated with low expression of programmed cell death ligand 1 (PD-L1) (8). Therefore, it is urgent to explore the cells in the TME and their effects on the response of immune checkpoint inhibitors (ICIs).

The tumor microenvironment (TME), which is made up of vessels, immune infiltration and extracellular matrix (ECM), promotes cancer growth, invasion and metastasis (9). Understanding the interplay between cancer cells and various immune cells in the TME such as T lymphocytes, dendritic cells (DCs), tumor associated macrophages (TAMs) and natural killer (NK) cells, could explain the pathogenesis and explore novel therapies for EOC (10) (11). Immune editing, defined as the dual function of the immune system, can suppress and/or promote tumor growth (12). Studying the dual function of immune cells in the TME can suppress the key pathways that inhibit antitumor responses, and promising therapies will be discovered (13). PD-1 and CTLA-4 expressed on T cells are the basis of immune checkpoint immunotherapy (14). Additionally, immunosuppressive molecules in the TME such as indoleamine-2,3-dioxygenase (IDO), interleukin-10 (IL-10) and prostaglandin E2 (PGE2), can also be targets of immunotherapies (15).

In this review, the effect of the TME in immunotherapies and progress in EOC immunotherapy will be discussed.

## 2 Tumor microenvironment

### 2.1 Suppressive immune cells

#### 2.1.1 Regulatory T cells

T-lymphocytes in the TME contain tumor infiltrating lymphocytes (TILs) and regulatory T cells (Tregs). Tregs have been shown to weaken antitumor immunity indicating poor prognosis in patients with EOC (16). Studies have revealed that increases in tumor Treg cells represent a low survival rate of EOC (16), while other studies show their association with a pleasing clinical outcome biomarker in colorectal cancer (17). Immunosuppressive mechanisms regulated by Tregs leading to immunological tolerance and ignorance of cancer are as follows: 1) releasing soluble or membranous repressive cytokines such as interleukin-10 (IL-10), interleukin-35 (IL-35) and transforming growth factor-β (TGF-β), which can kill effector T cells (18). 2) high expression of granzymes and perforin mediates the cytolysis of NK cells and cytotoxic CD8+ T cells (19). 3) interfering with effector T cell metabolism by reducing IL-2, which is competitively consumed by T cells, and increasing adenosine which is an inhibitory molecule (20). 4) inducing DC tolerance by expressing CTLA-4 and ligands CD80 and/or CD86 on DCs that can generate immunosuppressive tryptophan metabolites, lymphocyte activation gene 3 (LAG3) molecules can suppress MHC II molecules on DCs (21). In view of the key immunosuppressive effect of Tregs, several agents have been explored that directly target markers such as CTLA-4 and IL-2 (20, 22).

#### 2.1.2 Tumor associated macrophages

Tumor-associated macrophages (TAMs) are recruited from monocytes in blood and resident peritoneal macrophages, and these are major infiltrating immune cells in the TME (23). Given the important heterogeneity and plasticity, TAMs contain two groups: anti-tumorigenic M1 type and pro-tumorigenic M2 type (24). In the TME, the most pro-tumorigenic M2-like phenotype (25) is critical for cancer angiogenesis, invasion and metastasis through different kinds of cytokines, chemokines, growth factors, and proteases (26, 27). Vascular endothelial growth factor A (VEGF-A), a pro-angiogenetic chemokine and protease secreted by TAMs promotes tumor angiogenesis (26, 28). By producing epidermal growth factor (EGF), TAMs promote cancer cell proliferation (29). Moreover, TAMs exhibit immunosuppressive effects through secreting IL-10, TGF-β, CCL2 and arginase (30, 31). Current studies targeting TAMs mainly include: 1) suppressing M2-like TAMs *via* inhibiting the recruitment of TAMs and exhausting TAMs 2) activating M1-like TAMs by strengthening the repolarization of M2 macrophages into M1 macrophages (32, 33). For inhibition of the recruitment of TAMs, the CCL2/CCR2 axis barricade which is has been found to be helpful in a mouse ovarian cancer model (34), and disrupting the CXCL12/CXCR4 axis which prolongs the survival of a tumor mouse model (35) seem to be an encouraging therapy. The decrease in TAMs caused by inhibiting the CSF-1/CSF-1R pathway has been proven to strengthen the tumor suppressive effect of docetaxel (36). In EOC, trabectedin can effectively deplete macrophages by inducing apoptosis of macrophages and thus play an antitumor role in ovarian cancer (37). Paclitaxel treats ovarian cancer by reprogramming the M2 to the M1 phenotype through TLR4 in gene expression analysis (38). Furthermore, another macrophage-directed therapy targets PD-L1 on TAMs which is involved in tumor immune escape mechanisms (39, 40).

#### 2.1.3 Myeloid-derived suppressor cells

**Figure 1:**
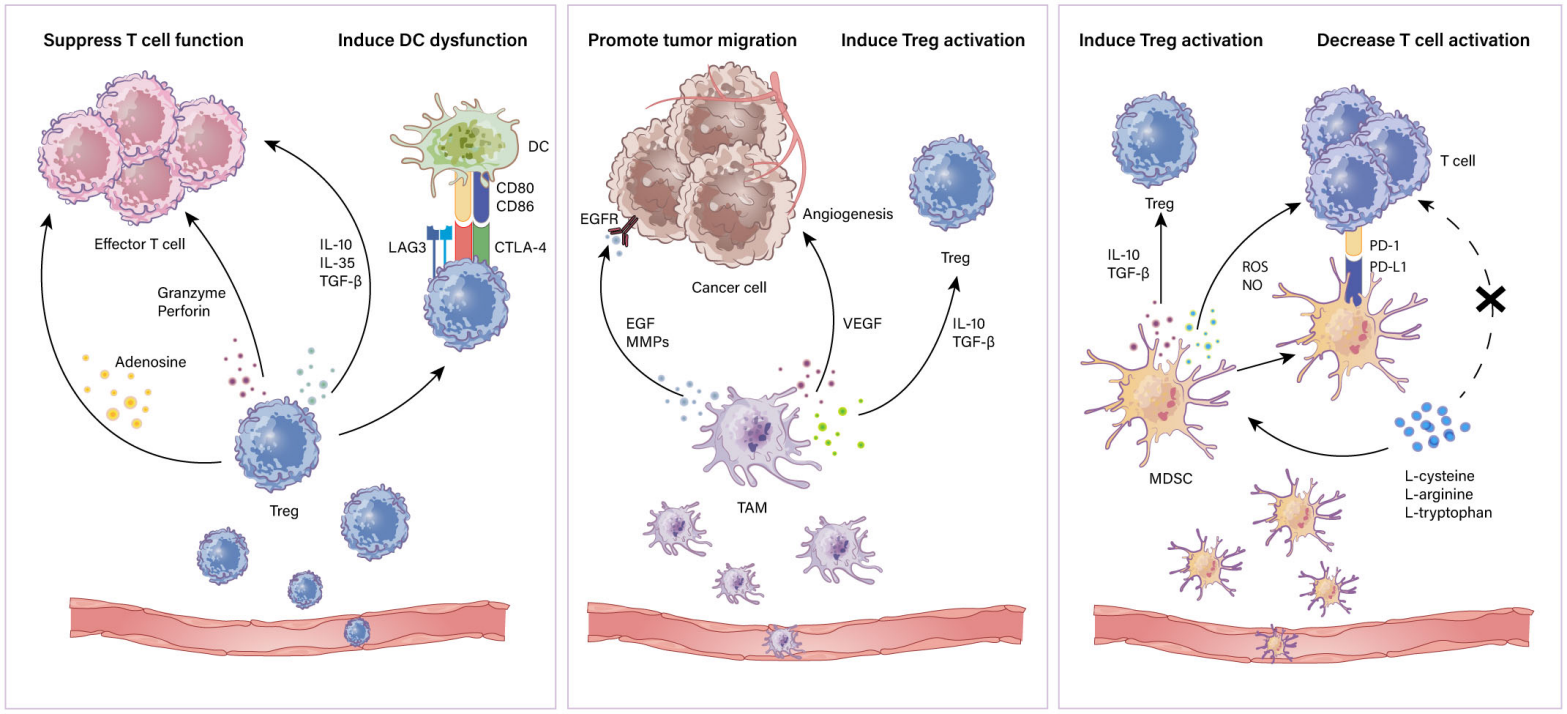
Immunosuppressive cells in the TME. Immune suppressive cells mainly contain Tregs, TAMs and MDSCs. Tregs restrain antitumor effector T cells including (i) secretion of repressive cytokines such as IL-10, IL-35 and TGF-β; (ii)induction of cytolysis *via* releasing granzymes and perforins; (iii) metabolic disturbance through adenosine production. Also, Tregs induce antigen presenting cell dysfunction for a tolerant phenotype. TAMs can (i) secrete EGF and MMPs to promote tumor progression; (ii) produce VEGF to aid in tumor angiogenesis; (iii) secrete IL-10 and TGF-β to induce Treg activation. MDSCs suppress T cells through (i) secretion of IL-10 and TGF-β which induce Tregs; (ii) production of NO and ROS which induce cytolysis and inhibit T cell activation; (iii) depletion of essential amino acids which play a crucial role in T cell activation and proliferation.

Myeloid-derived suppressor cells (MDSCs) are a subpopulation of immunoregulatory immature myeloid cells that increase in multiple pathologic situations (41). MDSCs have been implicated in suppressing T cells and impacting other cells in the TME (42). The mechanisms of suppressing T cells include the following: 1) accelerating the depletion of T cell essential amino acids such as L-cysteine, L-arginine and L-tryptophan which are critical for T cell activity (43–45). 2) expressing PD-L1 interacting with PD-1 on T cells to suppress the antitumor effect of T cells and thus promote immune evasion (46). 3) producing ROS and NO which are toxic to T cells (47). Moreover, MDSCs boost the activation of Tregs *via* IL-10 and TGFβ in the need of CD40 (48, 49). MDSCs can be targeted by various strategies: 1) reduction of MDSCs, 2) inhibition of the recruitment of MDSCs, 3) suppression of MDSC function and 4) induction of MDSCs to differentiate into non‐suppressive cells (50). Thus, immunotherapies in combination with targeting MDSCs could be a major strategy. Phosphodiesterase‐5 (PDE‐5) inhibitors targeting arginase 1 (ARG1) and iNOS restabilize the immunosuppressive response of T cells (51). Synthetic triterpenoids activate the Nrf2 gene which modulates antioxidant enzymes and nitroaspirin, inhibiting iNOS and ROS production and thus relieving the oxidative stress caused by MDSCs (52, 53). Furthermore, STAT3 inhibitors combined with immune checkpoint blockade have been shown to be beneficial in lymphoma (54). In addition, blocking COX‐2 which is correlated with the expression of ARG1 can also be a promising approach to attenuate MDSC function (55). A schematic illustration of how suppressive immune cells affect the antitumor immune response in the TME is shown in **Figure 1**.

### 2.2 Activated immune cells

#### 2.2.1 T lymphocytes

TILs contain CD8+ T and CD4+ T lymphocytes, especially CD8+ TILs which represent a good prognosis of EOC (56). CD8 + T cells recognize and kill pathogenic infections or cancer cells through perforin and granzyme (57). In addition to destroying cancer cells directly, CD8+ T cells suppress tumor vascularization *via* secreting IFN-γ which suppresses the development of cancer. Emerging evidence has revealed that CD8+ T cells in the TME are often beneficial to survival in ovarian cancer patients (58). Furthermore, CD4+ T cells are divided into different subtypes which include T helper 1 (Th1) cells, a group of cells that provide cytokines such as IL-2 and IFN-γ to support the antitumoral effect of CD8+ T cells (59). Moreover, high expression of CCL5 released by CD4+ T cells benefits the activation of DCs and thus induces an antitumor response (60). Hence, increases in Th1 cells within the TME are related to significant outcomes in a variety of cancers (61). As mentioned above, not all T cells function as antitumor effectors such as Tregs and Th17 cells. Thus, immunotherapies targeting the main impaired antitumor T effector cells are considered optimistic therapeutic approaches (62, 63). Recently, major advances have been made in developing PD-1 therapy which can potentiate the efficacy of CD8+ T cell based immunotherapy (64). T cells express PD-1 while other cells such as Tregs, TAMs, and cancer cells express PD-L1. Consequently, blockade targeting PD-1 or PD-L1 can suppress activation of T cell and breakdown immune tolerance and thus potentially mobilize immunity in tumors (65, 66).

#### 2.2.2 Natural killer cells

NK cells are the most efficient antineoplastic effectors and do not need any prior sensitization or HLA-independent tumor target recognition (67, 68). NK cells encompass two main populations with CD56bright/CD16– functioning to produce IFN-γ and TNF-α cytokines, while CD56dim/CD16+ killing tumor cells directly *via* releasing perforin/granzyme or through TRAIL pathways (69). However, NK cells usually exhibit dysfunction in the TME, such as reduced proliferation, decreased secretion of cytotoxic molecules and abnormal expression of immune checkpoints (70). Studies have revealed that PD-L1 on tumor cells can inhibit PD-1 expression on NK cells, thereby promoting immune escape from cancer (71). A series of molecules such as IDO and PGE2 secreted by fibroblasts were shown to suppress the expression of the activating receptor NKG2D and thus mediate immune escape (72). Several immunotherapeutic strategies based on NK cells are currently being explored including adaptive NK cells, cytokines, antibodies and ICIs (68). Remarkably, adaptive NK cell therapy induced by various cytokines exhibits enhanced antitumor effects in ovarian cancer (73). Currently, therapy based on cytokines such as IL-2 and IL-15 is shown to vigorously increase NK cells (74, 75). Although antibody-based immunotherapy is not the gold standard treatment for ovarian cancer patients, antigens on tumor cells including NY-ESO-1 and MUC1 have attracted great attention (76). Furthermore, evidence has revealed that PD-1 and CD96/TIGIT inhibitors potentiate the tumor lysis mediated by NK cells (77).

#### 2.2.3 Dendritic cells

**Figure 2:**
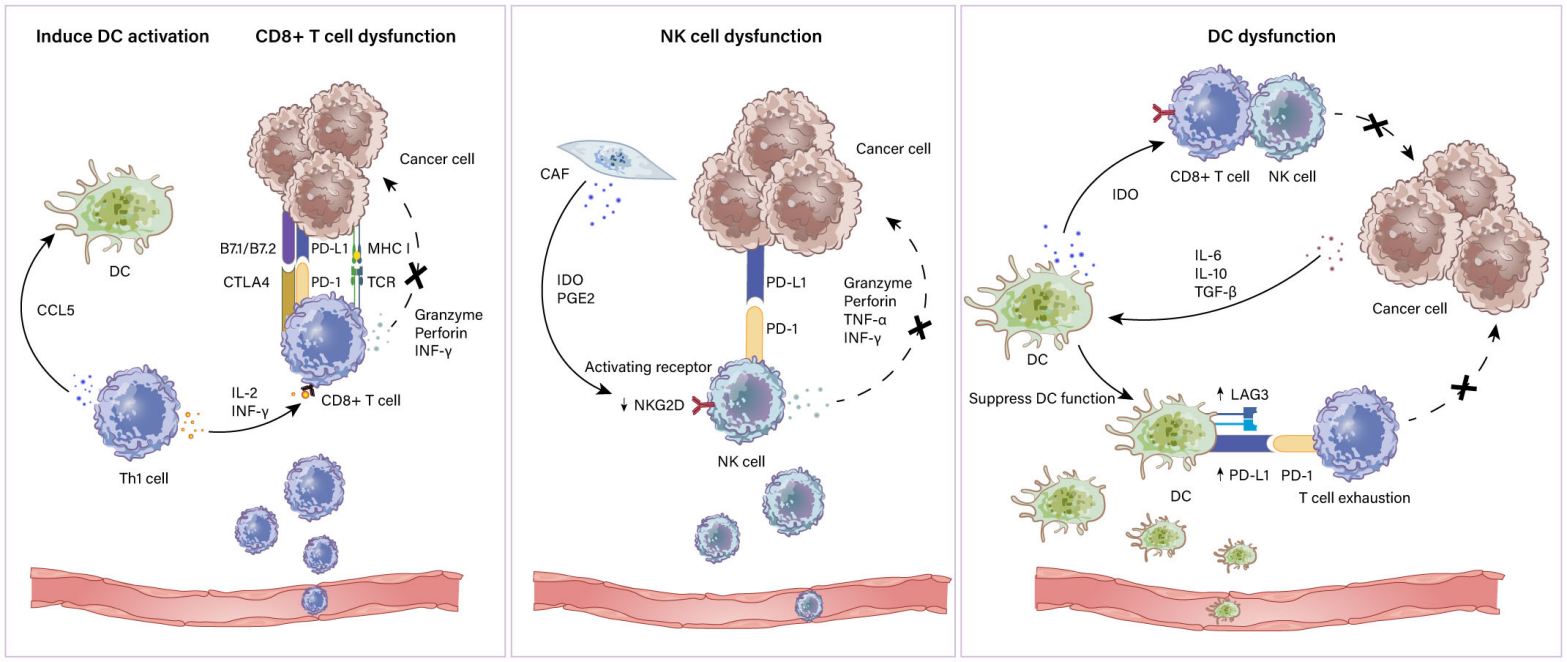
Immunoactivated cells in the TME. Immune activated cells include CD8+ and CD4+ T lymphocytes, NK cells and DCs. Th1 cell which is a subtype of CD4+ T cells express CCL5 to induce DC activation and produce IL-2 and IFN-γ to assist CD8+ T cells. CD8+ T cells recognize antigens expressed on MHC class I on cancer cells leading to the cytotoxic killing of cancer cells *via* granzymes and perforins. CD8+ T cell dysfunction is mediated by elevated inhibitory ligand PD-L1 and B7 molecules on cancer cells. NK cell dysfunction mechanisms include (i) PGE2 and IDO derived from CAFs downregulate the expression of activating receptor NKG2D on DCs; (ii) inhibitory ligand PD-L1 and B7 molecules expressed by cancer cells restrain NK cell function by combining with PD-1 and CTLA-4. The mechanism of impaired DC function includes (i) overexpression of PD-1 ligands and LAG3 on DCs results in T cell exhaustion; (ii) IL-6, IL-10 and TGF-β produced by cancer cells alter DC maturation and migration capacity.

DCs capture and process antigens to T cells and secrete inflammatory cytokines to induce pathogen-specific T-cell effects (78). Generally, DCs submit exogenous antigen peptides to CD4+ T cells through MHC II molecules and endogenous antigens to CD8+ T cells with MHC I molecules, and strengthen CD4+ and CD8+ T cell activity *via* presenting exogenous antigens (79, 80). However, the tumor microenvironment inhibits DC maturation through immunosuppressive factors including VEGF, IL-6, IL-10 and TGF-β, as well as repressive molecules including IDO (81, 82). Nevertheless, upregulating suppressive receptors such as PD-L1 and LAG3 induces T cell exhaustion thereby limiting the immune response (83). IDO produced by DCs restrains the function of NK cell and CD8+ T cell leading to an abated immune response (84). Therapeutic schemes targeting DCs have attracted great attention. Clinical studies revealed that IDO1 inhibitors combined with chemotherapy or ICIs elicit tumor regression (85). Collectively, potential DC based immune therapy seems to be an encouraging target against ovarian cancer. Furthermore, with such an effective ability for Ag presentation and T cell activation, DCs were tested as cancer vaccines that can produce “trained” DCs carrying tumor antigens and thus potentially induce strong antitumor T-cell effects (86, 87). In particular, it is well documented that DC vaccines combined with ICIs may result in synergistic effects (88, 89). A schematic illustration of how immune active cells exhibit dysfunction in the TME is shown in **Figure 2**.

### 2.3 Tumor-associated stromal cells

#### 2.3.1 Cancer-associated fibroblasts

CAFs derived from mesenchymal stem cells are crucial components of stromal cell types (90, 91). CAFs produce proteins, paracrine cytokines and various ECMs that contribute to shaping the tumor microenvironment (92, 93). Classical growth factors secreted by CAFs include the following: 1) TGF-β regulates the interaction between cancer and stroma thereby facilitating tumor initiation and progression (94). 2) Epidermal growth factor (EGF) maintains the expression of ATC integrin α5 (ITGA5) which can promote the early peritoneal spread of HGSOC (95). 3) Hepatocyte growth factor (HGF) contributes to proliferation *via* the c-Met/PI3K/Akt and GRP78 pathways (96). 4) CXCL12, IL6, and VEGFA induced by CAFs result in the epithelial-to-mesenchymal transition (EMT) which can promote peritoneal metastasis of ovarian cancer (97). 5) CAFs activate MMPs to assist in the growth, invasion, and metastasis of tumors (98). 6) Lipoma-preferred partner (LPP) which has been proven to increase the focal adhesion and stress fiber formation of ECs contributes to ovarian cancer chemoresistance (99). Therapeutic strategies targeting CAFs fall into several aspects, one of which involves TGF-β inhibitors which were shown to improve the overall survival of EOC in a mouse model with peritoneal metastasis (100). Imatinib, an inhibitor targeting PDGF-D produced by fibroblasts was found to suppress ovarian cancer cell growth (101).

#### 2.3.2 Cancer-associated adipocytes

Cancer-associated adipocytes (CAAs) are documented to promote stromal reshaping and the invasion of cancer cells through interacting with ovarian cancer cells in the TME (102). It has been proven that metastases appear in the omentum which is made up of adipocytes in most patients with ovarian cancer (103). Adipocytes secrete various molecules such as metabolites, MMPs, enzymes and growth factors supporting tumor cell progression (104). Furthermore, coculture studies found that CAAs boost the oxidation of cancer cells, indicating that CAAs supply energy to maintain the ovarian cancer cells proliferation (105). Additionally, CAAs produce adipokines that can promote tumor development. Among them, leptin stimulates the migration and invasion abilities of ovarian cancer cells through the JAK/Stat3, PI3K/Akt and RhoA/ROCK pathways (106). FABP4, a lipid chaperone protein is a key regulator in ovarian cancer metastasis (107). Molecules including IL-6, IL8 and TNF-α secreted by CAAs have also been proven to aid in the growth and invasion of breast tumor cells (108). Collectively, the reciprocation between CAAs and cancer cells results in the metastasis of tumor cells. Overall, therapeutic strategies specifically targeting lipid metabolism and transport such as FABP4 inhibitors in ovarian cancer are full of hope (107).

#### 2.3.3 Endothelial cells

Endothelial cells (ECs) impact the process of cancer growth and invasion (109). Angiogenesis is known to be a process regulated by the interplay of angiogenic activators and inhibitors. During tumor progression, oxygen deficiency and accumulation of metabolic products lead to hypoxia and acidity in the TME (110). Hypoxia in the TME induces the production of hypoxia-inducible factors (HIFs), which promote pro-angiogenic factors secreted by ECs, thereby promoting angiogenesis (111). In the course of angiogenesis, factors produced by cancer cells contain VEGF, platelet derived growth factor (PDGF), fibroblast growth factor 2 (FGF-2) and angiopoietins (112). VEGF, a chemokine secreted into malignant ascites contributes to the genesis of tumor blood *via* signaling with VEGF receptor-2 (113). To stabilize and increase the maturation of endothelial cell channels, pericytes express PDGFR-β which can interact with PDGF-B (114). Furthermore, FGF-2 promotes the production of MMPs, collagenase and plasminogen activator resulting in vascularization (115). In addition, FGF expression has been proven to be responsible for resistance to VEGF targeted therapies (116). Angiopoietin 1 and 2 (Ang1/2) has been found to promote the proliferation and survival of ECs *via* binding to the Tie-2 receptor (117). Thus, considerable attention is being paid to exploring therapeutic strategies to block the angiogenic signaling pathway. Bevacizumab as the most studied anti-VEGF monoclonal antibody has been demonstrated to positively increase PFS with cisplatin-based chemotherapy in several randomized phase III trials and has been recognized as the standard treatment in EOC (118, 119). Tyrosine kinase inhibitors (TKIs) such as pazopanib and cediranib are promising VEGFR targeting agents for ovarian cancer patients (120, 121). Trebananib, an inhibitor that targets non-VEGF signaling has meaningful effects on PFS when used in combination with paclitaxel in recurrent ovarian cancer through binding to Ang1/2 (122).

## 3 Novel combination approaches

It is obviously that ICIs have revolutionized immunotherapy and brought concrete benefits to many patients. However, the response rate is unsatisfactory with different anti-PD-1 or PD-L1 agents since EOC is known to have a high immunosuppressive TME and low expression of PD-L1 (8). Novel combination therapies are currently evolving. Inhibitor of PD-1 in combination with CTLA-4 increased the frequency of tumor infiltration by effector T cells as well as uniquely decreased the frequency of Tregs in tumors (123). Evidence from clinical trials found that this combination blockade therapy is effective. The NRG-GY003 study showed that in recurrent epithelial ovarian cancer, nivolumab combined with ipilimumab led to an effective response rate of 31.4% while nivolumab alone is 12.2%. In addition, the mPFS was 2 months in the monotherapy group and 3.9 months in the combination treatment group (124). Combining nivolumab with ipilimumab produced higher response rates and longer PFS in EOC than nivolumab alone, but is still limited, so more combination clinical studies such as NCT02834013 and NCT03508570 are underway.

In a mouse model of intraperitoneal ovarian cancer, compared with single drug therapy of AMD3100 (CXCR4 antagonist) and αPD-1, the antitumor efficacy of combined therapy in inhibiting tumor growth and prolonging the survival time of mice was significantly improved (35). This provides strong preclinical evidence for ovarian cancer combination therapy, but to date, no clinical trials related to ovarian cancer have been carried out. It is worth noting that in a phase IIa trial, disease control with BL8040 plus pembrolizumab was 34.5% in 29 patients. 22 patients were treated with BL8040 plus pembrolizumab plus chemotherapy. The results exhibited an effective response rate of 32% and a disease control rate of 77% (125). These data all suggest that the combination of CXCR4 antagonists and PD-1 inhibitors can amplify the antitumor effect of chemotherapy.

In recent years, transmembrane protein triggering receptor expressed on myeloid cells 2 (TREM2) has gained great attention in anti-PD-1 resistant therapy since its enriched expression on TAMs in EOC (126). In a model of invasive ovarian cancer *in situ*, anti-TREM2mAb therapy can drive effective antitumor immunity (126). This finding indicates that TREM2 is a potential immunotherapy target when ICIs are ineffective and TAMs are rich in the tumor microenvironment. A phase I clinical trial (NCT04691375) to evaluate the single drug anti-TREM2 and anti-TREM combined with pembrolizumab in solid tumors which include ovarian cancer is underway.

Evidence has shown that PD-1 blockade in combination with a VEGF-A inhibitor can potentiate antitumor efficiency *via* increases in CD8+ and CD4+ T cells, and decreases in MDSCs and Tregs (127, 128). In a single-arm phase 2 clinical study, 38 women with recurrent epithelial ovarian cancer were screened for intravenous treatment with nivolumab and bevacizumab. The results showed that the ORR was 28.9%, among which 40.0% were platinum sensitive patients and 16.7% were platinum resistant patients (129). These data indicate that the combining nivolumab with bevacizumab is effective and feasible in ovarian cancer patients, especially those who are sensitive to platinum.

Poly-(ADP)-ribose polymerase inhibitors (PARPi), known as synthetic lethal agents in tumors with BRCA1/BRCA2 mutations are rapidly evolving (130). Combining PARPis with PD-1/PD-L1 blockades is a promising therapy that can synthetically enhance the antitumor effect (131). A phase II clinical study of MEDIOLA found that 32 patients received olaparib combined with durvalumab, and the overall disease control rate was 81% (132). The final result of the study showed that the OS of 31 patients treated with olaparib combined with durvalumab and bevacizumab was 31.9 months compared with that of 23.2 months in the two-drug group. In addition, the DCR of the two-drug group at 56 weeks was 9.4%, and that of the three-drug group was 38.7%, which indicated that the three-drug treatment mode was superior to the two-drug treatment mode for platinum-sensitive recurrent non-gBRCA ovarian cancer patients (133). The three-drug regimen has been applied to a third-phase clinical study of first-line maintenance therapy (NCT03737643).

## 4 Discussion

Immune checkpoint immunotherapy has been the most prominent therapeutic strategy for successfully treating different kinds of cancers. However, the response rate in EOC is low since the immunosuppressive tumor microenvironment could limit the efficiency of ICIs. It is urgent to improve the effect of immunotherapy for EOC. Novel targets have been described and targeting approaches combined with ICs have already impacted the clinical outcomes of ovarian cancer. Targeting immune subtypes such as TAMs, Tregs, CAFs or angiogenesis could contribute to potentiating the antitumor effect of ICs. However, there are still many details to explore and discuss. In summary, the constituents within the TME should all be considered to explore novel combinations that contribute to achieving maximal benefits in EOC.

## Author contributions

YW and LZ wrote the original manuscript. YB drew the figure. XM and LW corrected the original draft. All authors contributed to the article and approved the submitted version.

## Conflict of interest

The authors declare that the research was conducted in the absence of any commercial or financial relationships that could be construed as a potential conflict of interest.

## Publisher’s note

All claims expressed in this article are solely those of the authors and do not necessarily represent those of their affiliated organizations, or those of the publisher, the editors and the reviewers. Any product that may be evaluated in this article, or claim that may be made by its manufacturer, is not guaranteed or endorsed by the publisher.

## Abbreviations

EOC, epithelial ovarian cancer; TME, tumor microenvironment; PD-1, programmed cell death 1; PD-L1, programmed cell death ligand 1; ECM, extracellular matrix; DCs, dendritic cells; TAMs, tumor associated macrophages; NK, natural killer; ICIs, immune checkpoint inhibitors; Tregs, regulatory T cells; IL-10, interleukin-10; IL-35, interleukin-35; TGF-β, transforming growth factor-β; IDO, indoleamine-2,3-dioxygenase; PGE2, prostaglandin E2; LAG3, lymphocyte activation gene 3; VEGF-A, vascular endothelial growth factor A; EGF, epidermal growth factor; MDSCs, myeloid-derived suppressor cells; ARG1, arginase 1; CAFs, cancer associated fibroblasts; CAAs, cancer-associated adipocytes; HGF, hepatocyte growth factor; PDGF, platelet derived growth factor; FGF-2, fibroblast growth factor 2.
